# Challenges of NGO-to-state Referral in the Delivery of HIV Prevention Programs in Ukraine Supported by the Global Fund

**DOI:** 10.5195/cajgh.2015.213

**Published:** 2016-03-29

**Authors:** Svetlana McGill

**Affiliations:** Queen Margaret University, Edinburgh, United Kingdom

**Keywords:** HIV care continuum, Global Fund, linkage to care, NGOs

## Abstract

**Background:**

Ukraine has one of the world’s fastest growing HIV rates and was one of the largest recipients of funding from the Global Fund to Fight AIDS, Tuberculosis and Malaria (GF). The objective of this study was to close the gaps in the literature on the delivery of HIV prevention services by NGOs and the perceptions of NGO delivered services, using as an example HIV prevention programs in Ukraine funded by the GF.

**Methods:**

The aim of this qualitative study was to determine how NGO-based services were implemented in the context of a state-owned healthcare system of Ukraine. An ethnographic study, which included 50 participant interviews, was conducted in three oblasts in Ukraine and in the capital, Kyiv, between 2011 and 2013. This article presents some of the findings that emerged from the analysis.

**Results:**

Participants reported that NGOs were focused more on reporting numbers of rapid tests, and less on motivating clients to continue onto treatment. The role division between NGOs and the state in HIV services was largely perceived by participants as unclear and challenging. Overall, lack of clarity on the role of government healthcare providers and NGOs in providing HIV services compromised the process of finding, referring, and retaining HIV patients in care.

**Conclusions:**

Gaps in linking HIV patients to the HIV care continuum have been identified as a potentially problematic issue in delivery of HIV prevention services by GF funded NGOs. With an anticipated GF exit from Ukraine, the lack of clearly defined NGO-to-state referrals of HIV patients complicates the transition of NGO run services into state funding. Further steps to improve referral systems are necessary to ensure a smooth transition and enable Ukraine to fight its HIV epidemic effectively.

Since 2008, HIV rates have steadily increased in Eastern Europe and Central Asia.^1^ Russia and Ukraine account for over 90% of diagnosed HIV cases; however, Ukraine has the most severe increase among the Eastern European and Central Asian countries, with an estimated 440,000 cases of people living with HIV^2^ and an estimated HIV prevalence of 1.63% at the end of 2007.^3^ As of February 11, 2014, there were 247,101 registered cases of HIV infection, over 66,607 registered cases of AIDS, and 32,283 AIDS deaths.^4^ Recent political turmoil has led to claims that the Ukraine’s AIDS program is “breaking down” and international organizations are concerned that HIV rates are beginning to rise for the first time since 2002.^5^

Ukraine has been among the top ten recipient countries of the Global Fund (GF) to Fight AIDS, Tuberculosis, and Malaria. In 2003, Ukraine received a GF Round 1 (R1) HIV grant of 95 million USD. The principal recipients were the Ministry of Health (MOH), United Nations Development Programme, and the Ukrainian Fund against HIV/AIDS. Utilization of the grant funds quickly stalled primarily due to inefficient governance and slow program implementation.^6^ As a result, in 2004, GF suspended the funding to all recipients and transferred the R1 grant to an international nongovernmental organization, International HIV/AIDS Alliance, based in the United Kingdom. In 2012, a grant was awarded to two NGO principal recipients.

An anticipated outcome of the GF decision to transfer funding to an international nongovernmental organization was the creation of robust and effective delivery of HIV services. With the MOH as an original implementer, the services were perceived to be delivered by state healthcare, the backbone of which was the network of specialized regional AIDS clinics. Ukraine’s healthcare is often viewed as a “hybrid Semashko” system^7^ because many elements of the previous Soviet healthcare system are still present. A network of AIDS clinics, known as AIDS centers, represents one such element. After control of GF funds was transferred to an international nongovernmental organization, the perceived division of roles between state healthcare and NGOs ceased to exist, bringing implementation challenges to Ukraine’s original GF program, which was geared to state healthcare.

## HIV care continuum and perceived NGO delivery roles in Ukraine

For individuals with HIV infection to fully benefit from antiretroviral therapy, they need to know that they are HIV infected, be engaged in regular HIV care, and receive and adhere to effective antiretroviral therapy.^8^ ‘The HIV care continuum’ -- also known as ‘the HIV treatment cascade’ -- is a model used to describe the delivery of HIV services to people living with HIV across the entire continuum of care.

In Ukraine, a distinction is made between “testing to identify HIV” - typically done through rapid HIV tests at a variety of settings – and “making an HIV diagnosis” (or confirmatory test) – that includes other assessments/testing and can be performed only at healthcare facilities.^9^ Importantly, administration of antiretroviral therapy and other free HIV continuum healthcare services associated with an HIV positive test begin when a patient presents with a positive HIV result obtained from confirmatory testing for antibodies to HIV and antigen р24 HIV-1.^10^ The patient is then put on a dispensary list, which requires registration and submission of individual passport data to the AIDS center. Thus, confirmatory HIV testing is important to link the patient to Ukraine’s HIV healthcare continuum. Ukraine implements the combined voluntary counseling and testing model, administered through an extensive, tiered HIV laboratory system in state-funded hospitals, sexually-transmitted disease (STD) clinics, narcological and tuberculosis dispensaries, family planning, and antenatal clinics.^11^ Virologic and immunologic testing is performed at over 27 regional AIDS center laboratories, as well as at a central HIV reference laboratory. Some 761 state-funded Dovira (Trust) centers, located in residential areas in all oblasts (or states) in Ukraine,^12^ conduct express testing and counseling. Rapid tests are also administered by HIV-service NGOs.^11^ An evaluation of the GF implementers in Ukraine by Australian AIDS Projects Management Group underlined that “successful referral from testing to treatment is key to controlling the epidemic,” but noted that “the division between the sectors and lack of government resources means that many people who test positive do not get treatment” and that the linkages between relevant NGOs and government services were “highly variable.”^13^ Eight years after the GF began supporting NGO provision of HIV services, a USAID funded publication found that a “licensing and accreditation system has not actually been developed or implemented.”^14^ Lack of a legal framework carried the risk that many NGO-based health services would have to be provided *ad hoc* and, as such, might not be recognised by the state.

While the HIV care continuum model appears to be well-established in many countries across the world, research into practices bringing people living with HIV into the healthcare continuum is insufficient in Ukraine. The objective of this study was to close the gaps in the literature on the delivery of HIV prevention services by NGOs and the perceptions of NGO delivered services, using as an example HIV prevention programs in Ukraine funded by the GF.

## Methods

Consistent with a significant body of research of aid programs in post-communist countries,^15^–^19^ an ethnographic study design was chosen as the most appropriate research approach in this setting.

### Data collection

Primary data were collected between 2011 and 2013 through 50 in-depth, open-ended, face-to-face interviews with purposively selected participants with experience in GF programs, based in Kyiv, and in three oblasts of Ukraine. An interview guide was developed for use in conducting the interviews. The interview guide structured inquiry into the following open-ended questions:

1. Principal Recipient NGOs roles and relations with other healthcare actors in GF programs;
2. Linkage of HIV services provided by GF funded NGOs with state healthcare; and
3. Focus on GF funded HIV prevention services.

In addition, literature and documents were searched about HIV services provided by NGOs using PubMed and Google Scholar. Conceptualizations from the literature were synthesized with the findings that emerged from the interview data analysis.

Secondary data analysis included review of GF program documents such as principal recipient annual reports, minutes of Country Coordinating Mechanism (CCM) and stakeholder meetings, PR and government press releases, documents on the web-sites of the GF, State Service of Ukraine for AIDS and Other Infectious Diseases, the Ukrainian Center for Disease Control, and two principal recipients.

### Respondents’ sampling criteria and setting

**Figure 2.**
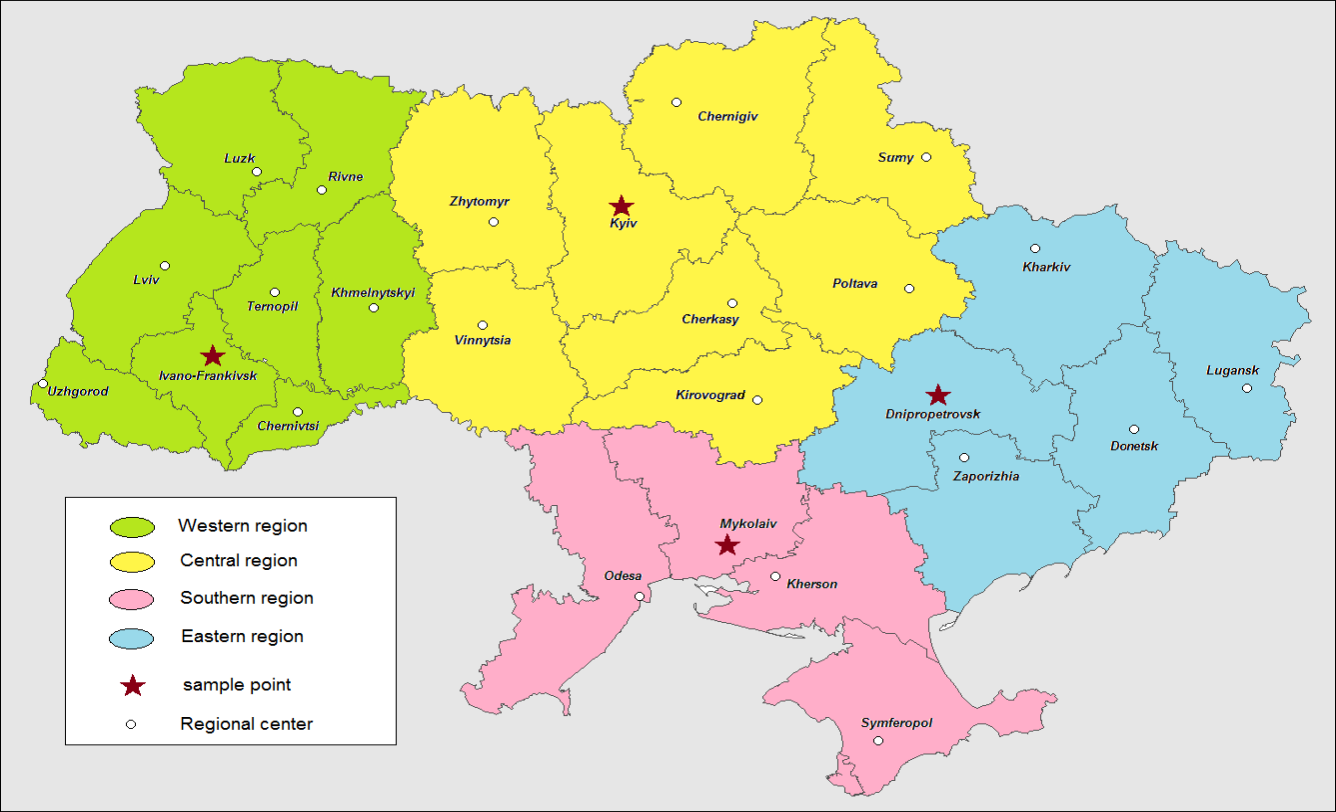
**Location of the sampling points (MapInfo 7.0)**

A key criterion for inclusion into this research was the respondent’s experience of engagement with GF program implementation. Following a review by the Queen Margaret University Ethics Research Panel, ethical approval was obtained in order to conduct the interviews. Respondents were primarily national and regional government stakeholders, NGO service providers, and state healthcare service providers, purposively selected to have an experience of previous or current engagement with the Global Fund to Fight AIDS, Tuberculosis and Malaria program implementation. At the oblast level, key informant interviews were conducted with local government and health officials and staff of GF sub-recipient NGOs. The respondents were also categorised into three geographical levels according to their location: sub-national (region), national, and international. The choice of oblasts was meant to reflect on the regional balance of Ukraine, diffusion of its HIV epidemic, and the perceived depth of penetration by the the Global Fund to Fight AIDS, Tuberculosis and Malaria programs. Figure 2 represents the locations of the sampling points.

**Figure 3.**
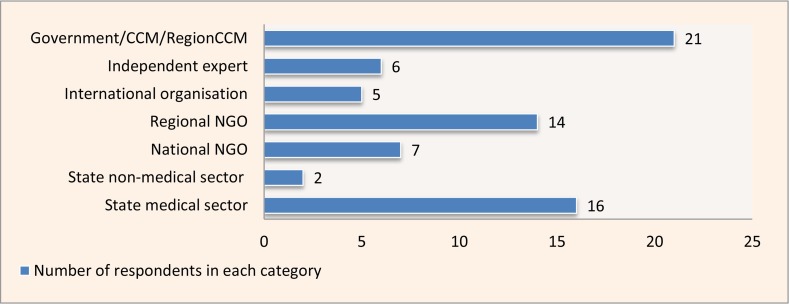
Self-identification of participants* *Note. Country Coordinating Mechanism (CCM) membership was reported in addition to the main respondent category. Therefore, the total number of respondents is above 50.

Twenty-seven interviews were conducted in oblasts, and 23 interviews were conducted with national and international stakeholders. Respondent’s sector identification was determined through self-assessment (Figure 3). The majority of the participants (72%) had five or more years of experience with GF programs, and only 8% of participants had less than 2 years of experience.

### Data analysis

The interviews were transcribed verbatim, then coded and analyzed using thematic content analysis. The steps included matrix-based categorization of data from the interviews and theoretical coding in which open codes and categories were compared to generate an analytic schema to interpret the findings. In line with an ethnographic inquiry paradigm, the analysis attempted to capture as many accounts as possible within the chosen thematic categories, yielding broad accounts of various aspects of GF implementation. Following social science practices of data representation,^20^ verbatim quotations from participant interviews are used widely in this paper as evidence for the author’s interpretations, and for illustration purposes. Quotations are presented using the numbers rather than sector or regional identities in order to protect participants’ anonymity.

## Results

The sections below outline the results that emerged from the analysis based on the interview data collected from 50 respondents.

### (1) Linkage of HIV services with state healthcare

Following features characterized HIV services provided by NGOs:

(1a) A ‘broken link’ in the chain of HIV services
  Participants reported that NGOs were focused more on reporting numbers of rapid tests, and less on motivating clients to continue onto treatment (to illustrate, participant verbatim quotations are provided in Appendix 1).
(1b) Inconsistent referral practices
  Among participants overall, there was no uniform view of what constituted a successful referral. While some participants understood a successful referral as linking a patient to an official registration, others believed that only the retention of a patient in care constituted a full referral (see section 1b in Appendix 1). As to the reasons for inconsistent referral, respondents noted the following: (1) the absence of referral protocols, (2) ineffective client management, and (3) lack of patients. Some respondents doubted the existence of referrals and suggested there were no client referral services in Ukraine at all.
(1c) Use of coupons to regulate referral
  Respondents identified the following problems about the coupon system: scarcity of coupons, number of coupons limited per day, and overburdened staff at AIDS clinics (see section 1c of Appendix 1).

### (2) Gaps in GF funded HIV prevention services

A lack of focus on a confirmatory HIV testing was demonstrated by primary data analysis. Study participants reported that GF funded activities appeared to be more concentrated on the early ‘field stage’ of finding a client and providing a rapid test, rather than on follow-up to care. Participants reported that the focus on rapid tests conducted by NGOs led to services not being seen as part of state healthcare, nor being counted in state statistics (see section 2b in Appendix 1).

### (3) Role division between NGOs and state and sustainability in GF programs

The role division between NGOs and state in HIV services was largely perceived by participants as unclear and challenging, and it affected the division of labor between them as actors in service delivery. The NGOs’ role as GF implementers was also described as contradictory. Unclear role division between GF funded NGOs and state could be a reflection of different views between GF and state on HIV prevention (see section 3 in Appendix 1).

## Discussion

This study is one of the first qualitative studies examining the delivery of NGO HIV services in GF funded programs in Ukraine. The study results suggest disconnect in the delivery of HIV services by GF funded NGOs, and identified gaps in linking HIV patients to the HIV care continuum.

Literature suggests that the linkage between GF funded services and state healthcare in Ukraine should be comprised of providing services to vulnerable communities and to link clients to needed services.^21^ When NGO referral practices to AIDS centers were reviewed, there was no follow-up conducted to ensure that the client engaged with the state services, indicating that HIV positive clients may be lost to follow-up.^22^ In addition, several publications noted a lack of consistent referral practices between NGOs and government services.^13^,^23^ These studies analyzed HIV services provided to the people who inject drugs, concluding that client referrals were “inconsistently applied and frequently consisted of informal sign posting rather than formalised referral across government and NGO providers.”^13^ These findings are consistent with the data collected from the interviews. Participants reported that the NGOs spent little effort on ensuring clients continued treatment, developing consistent referral protocols, and effectively transitioning clients from one stage of HIV care to the next.

An analysis of published PR documents demonstrated that in Round 6 (R6), in response to concerns about the referral process, the principal recipients established a system of referring people who tested HIV positive using rapid tests by NGOs by providing them with *talony* -- appointment coupons to undergo confirmatory testing with an AIDS clinic. This procedure was also mentioned by Varban et al.^11^ Interview respondents attested to a scarcity of coupons and daily limits on coupons, which may indicate a need to ration access to confimatory testing due to the number of rapid HIV tests conducted by NGOs generating more potential HIV carriers than the state sector’s screening capacity was prepared to handle.

Confirmatory HIV testing, and not rapid HIV screening, is deemed important for the purpose of linking patients with appropriate care canters in Ukraine. Recent publications have identified three major gaps associated with rapid HIV screening: (1) failure of clients from high-risk groups to be identified at an early, rapid HIV screening stage “to return to the AIDS Center to receive their confirmatory test results;”^13^ (2) sub-recipients “not providing incentives to clients to pick up the results of the tests” from the AIDS Center;^13^ (3) legal constraints – by Ukrainian law, HIV screening by rapid tests can only be executed by the medical staff of state institutions, who also have the exclusive right to communicate test results to the patients.^11^ Similarly, Judice et al.^14^ noted regulatory gaps remaining in NGO run mobile units, also funded by the GF, such as the requirement to sub-contract a doctor to inform the patient of the HIV screening results, and the inability of NGOs to provide clients with official certification – *spravka* – of the test results. In addition, the delivery of HIV prevention services between governmental and non-governmental service providers appear to remain poorly coordinated, which presents “a risk to the sustainability of prevention programs currently supported by the Global Fund grants and the viability of overall national prevention efforts.”^24^

Participant responses and analyzes of published data both suggest that GF funded HIV programs only partially fulfilled the objective of improving the National AIDS Prevention, Treatment and Support Program. While GF funded HIV services appeared to be more focused on outreach, field-based *(polevye)* activities, such as distribution of commodities (syringes, condoms, or information brochures) and on preliminary, rapid HIV screening, linkage to the next stages of HIV care continuum appeared weak, with no traceable follow-up for a confirmatory screening with state AIDS clinics, or entry and registration into antiretroviral therapy and other treatment. NGO referral systems varied among different NGOs and were mostly *ad hoc* with no referral protocol or coordination to track entities that provided services to clients. Lack of effective referrals meant that fewer patients could enter the HIV care continuum or receive antiretroviral therapy. The lack of clearly defined referral standards to facilitate referral of HIV patients from NGO services into the state care may impair Ukraine’s ability to fight its HIV epidemic effectively.

There is an urgent need to balance the numbers of HIV positive individuals identified by NGOs with the ability of AIDS centers to provide HIV care that requires better defined referral standards, effective strategy of NGO-to-state referral, and an increased ownership of *oblasts* over local re-programming of GF funding. With an anticipated GF exit from Ukraine in 2017, NGO run HIV services would need to transit into state funding. Further steps to improve referral systems are necessary in order to enable Ukraine to assume full ownership of its health programs and to manage its HIV epidemic effectively.

Results of this study question some of the existing views of civil society organizations as central in providing HIV services in conditions of insufficient, scattered, or even non-existent state healthcare settings that is typical in many regions where HIV is highly prevalent. Role division between state healthcare providers and NGOs in provision of HIV health services resulted in an accountability gap.^25^ While the NGOs received large funding from GF, they were not legally obligated to bring clients into care, while government providers had an obligation to provide care but did not have the funding, as reported by participants. As a result, the capacity of Ukraine’s post-Semashko healthcare to provide treatment for HIV patients appeared compromised by unclear boundaries between NGO run prevention services and state AIDS clinics, resulting in gaps in the process of finding, referring, and retaining HIV patients in care.

### Study strengths and limitations

While this is one of the first studies conducted to evaluate HIV GF programs in Ukraine, it should be noted that this ethnographic study is limited in scope and does not evaluate the impact of such programs on the national level. The sample size and location of data collection points, while in line with qualitative research standards, may not be sufficient to generate generalized conclusions on the impact of GF programs to the whole country. This study has important implications for future research in this area, as it raises a number of problems and gaps in existing publications that may fuel further interest in researching GF programs in Ukraine. The analysis and the discussion presented in this paper are based on the research that was completed prior to later changes in the GF grant systems in 2012 and before the 2014 economic and political crisis in Ukraine occurred. The major strengths of this study include analyzing a very important under-investigated problem in Ukraine and conducting interviews in multiple locations, thus improving generalizability of research findings.

## Figures and Tables

**Table 1.** A model of HIV treatment cascade^8^

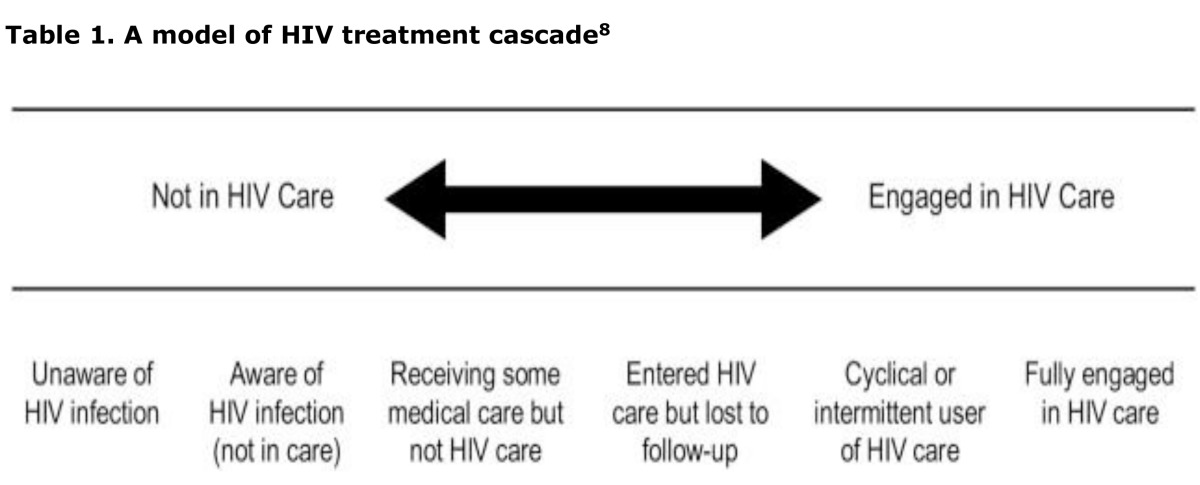

## Appendix 1. Participants’ views on NGO-to-state linkage practices in GF-funded programs

**Table:**

Emerging themes	Participant characterizations	Verbatim illustrative quotes
(1): Linkage of HIV services with state healthcare	*(*1a) A ‘broken link’ in the chain of HIV services	NGOs report numbers… but how to get people to start going to... get tested, how to stop them from being afraid of testing, and how to help those people who are found to have HIV become less afraid of getting treatment (009: 92–94)
	(1b) Referral practices: Inconsistent	“[During] referral to confirmatory tests, coverage sharply falls.” (040: 299)
	Successful referral means:	
	- linking a patient to official registration:	There is a need to work continuously with a person who has received a positive result until he or she gets registered (028: 223).
	- only when patient is retained in care	Here they found a drug user, took him to [get] methadone…There they provided him with information and counselling. But the final goal is not just finding a person, or even bringing him for treatment. It is retaining him there (020: 441–443). You not only need to count services…You need to take the patient to the logical end. (019: 100)

	Reasons for inconsistent referrals:	
	- absence of referral protocols	“There are many NGOs around the AIDS center, but no referral protocol” (013: 329–330);
	- ineffective client management	“There is no client base. Clients may enter programme several times” (019: 571–586); “Client management is not well developed, there is lots of subjectivity” (018: 125–127);
	- lack of patients	“In 2012, the government and the GF doubled the number of patients – to have twice as much more patients on antiretroviral therapy... but suddenly they realised that they could not find the people. First, there were not enough drugs, now there were enough drugs, but they could not get the people”. (050: 35–40)

	Client referral did not happen	‘no client referral services in Ukraine at all’ (012: 377), (014:24).

	*(*1c) Practices of using coupons to regulate referral	“the coupons were “scarce, hard to get” (019: 344–345) “the number of coupons was limited per day” (032: 342–343).
	The coupon appointment system overburdened the AIDS center staff	Our *oblast* AIDS center is suffocating. Our NGO takes 7 out of 20 coupons that are for an AIDS center visit. The remaining 13 coupons go to other NGOs. It means that an AIDS center can only receive 20 people per day, which means 100 people per week. They are suffocating... Staffing is a problem (019:286–288).

(2) Gaps in GF-funded HIV prevention services	2(a) Services concentrated more on early ‘field’ stage of finding a client, less on linking him or her to care:	“The lion’s share of the money is being spent on finding clients… when instead, it should be spent on retaining [them] in healthcare…they [NGOs] are fixated on field work… they spend so much money in the field… but their work ends there. Clients do not reach the treatment stage” (020: 443–446).
	2(b)‘rapid HIV tests by NGOs are neither viewed as part of state healthcare, nor counted in state statistics	“A quick test is not confirmatory…People need to go to an AIDS centre to have the second test.” (028: 216–217) “The MOH does not report quick screening by NGOs. It only reports on the tests they have done.” (029: 232–233) “You can pass a quick test a million times, but you are nobody for the [health] system. You only become a patient after a confirmatory screening” (040: 299–300).

(3) Role division between NGO and state services	Unclear, challenging	There is no clear division of roles…What is state doing? What are NGOs doing?... We need to define this division clearly... and then we won’t interfere in their work and they won’t interfere in ours (020: 352–358). We do not want to take over the roles that the state should fulfil - treatment, or prevention... we cannot substitute the state. (036: 158–180)
	Contradictory	There is a discrepancy in that the government is responsible for prevention, but implementation rests with NGOs... There is a contradiction here... (049: 60–66).
	Based on different views between GF and state on HIV prevention:	There is a big contradiction that large funds are concentrated with NGO but government is responsible for healthcare. The risk is that money will not be spent for the purpose it needs to be spent, because GF priorities in funding [HIV] prevention may not coincide with state policy or even run counter to it. (025: 267–270)

	NGO services are unsustainable	The state is used to NGOs doing prevention. But if [GF] funding stops, the state is not ready to support this work. It is only loyal to NGOs because they receive grants. If the state has to fund this, it will not. It has other priorities. (036: 204–231)
